# SOX7 inhibits the malignant progression of bladder cancer via the DNMT3B/CYGB axis

**DOI:** 10.1186/s43556-024-00198-8

**Published:** 2024-09-04

**Authors:** Jingcheng Zhang, Wentao Zhang, Ji Liu, Yuchao Liu, Yufeng Jiang, Ailiyaer Ainiwaer, Hanyang Chen, Zhuoran Gu, Haotian Chen, Shiyu Mao, Yadong Guo, Tianyuan Xu, Yunfei Xu, Yuan Wu, Xudong Yao, Yang Yan

**Affiliations:** 1 Department of Urology, School of Medicine, Shanghai Tenth People’s Hospital, Tongji University, Shanghai, China; 2 Urologic Cancer Institute, School of Medicine, Tongji University, Shanghai, China; 3 Department of Urology, Chongming Branch, School of Medicine, Shanghai Tenth People’s Hospital, Tongji University, Shanghai, China; 4 Department of Urology, Xinjiang Uygur Autonomous Region, Kashgar Prefecture Second People’s Hospital, Kashgar, China; 5 Department of Urology, Hefei Cancer Hospital, Chinese Academy of Sciences, Hefei, China

**Keywords:** Bladder cancer (BCa), Sex determining region Y-box 7 (SOX7), Methylation, Cytoglobin (CYGB)

## Abstract

Bladder cancer (BCa) stands out as a highly prevalent malignant tumor affecting the urinary system. The Sex determining region Y-box protein family is recognized for its crucial role in BCa progression. However, the effect of Sex determining region Y-box 7 (SOX7) on BCa progression has not been fully elucidated. Herein, RNA-sequencing, western blot (WB), immunohistochemistry (IHC), immunofluorescence (IF) and tissue microarray were utilized to assess SOX7 expression in vitro and in vivo. Additionally, SOX7 expression, prognosis, and SOX7 + cytoglobin (CYGB) score were analyzed using R software. In vitro and vivo experiments were performed with BCa cell lines to validate the effect of SOX7 knockdown and overexpression on the malignant progression of BCa. The results showed that SOX7 exhibits low expression in BCa. It functions in diverse capacities, inhibiting the proliferative, migratory, and invasive capabilities of BCa. In addition, the experimental database demonstrated that SOX7 binds to the promoter of DNA methyltransferase 3 beta (DNMT3B), leading to the transcriptional inhibition of DNMT3B. This subsequently results in a reduced methylation of CYGB promoter, ultimately inhibiting the tumor progression of BCa. SOX7 + CYGB scores were significantly linked to patient prognosis. In conclusion, SOX7 inhibits the malignant progression of BCa via the DNMT3B/CYGB axis. Additionally, the SOX7 + CYGB score is capable of predicting the prognostic outcomes of BCa patients. Therefore, SOX7 and CYGB may play an important role in the progression of bladder cancer, and they can be used as prognostic markers of bladder cancer patients.

**Supplementary Information:**

The online version contains supplementary material available at 10.1186/s43556-024-00198-8.

## Introduction

Bladder cancer (BCa) ranks among the most prevalent malignancies of the urinary system, with around 580,000 newly diagnosed cases worldwide in 2020 [1]. According to estimations by the World Health Organization, this number is expected to double by 2040 [2]. About 75% of BCa is non-muscle invasive BCa (NMIBC) [3]. The primary treatment approach involves bladder-preserving transurethral resection of bladder tumors combined with postoperative bladder infusion chemotherapy [4, 5]. Despite these treatments, the 5-year recurrence rate of such individuals remains as high as 50%-70%, with 10%-15% of patients progressing to muscle invasive BCa (MIBC). Every year, about 25% of new cases of BCa are categorized as MIBC, with a 5-year tumor-specific survival rate of around 50%. Platinum chemotherapy stands as the primary treatment strategy for advanced BCa; however, more than 50% of patients do not respond favorably to chemotherapy [6]. Following chemotherapy, advanced BCa patients who continue to progress or relapse typically face an average survival period of merely 12–14 months [7]. In addition, BCa has a high rate of progression and recurrence [8], underscoring the urgent need to elucidate the molecular mechanisms governing the malignant progression of BCa.

Various SOX (Sex determining region Y-box) proteins have been acknowledged for their role in disease development. More recently, they have gained recognition as important contributors to the onset and progression of human cancer [9]. Sex determining region Y-box 7 (SOX7) has been recognized as a member of the SOX family. Initially cloned by Shiozawa M in African Xenopus and mice [10, 11], it exerts a crucial function in development and endodermal differentiation. The human SOX7 gene is located on chromosome 8p23.1 and encodes 377 amino acids [12], comprising an intron and two exons. SOX7, SOX17, and SOX18 belong to the SOXF subfamily [13, 14]. Like other SOX factors, the N-terminus of SOXF factors contains highly conserved HMG DNA domains and transcriptional activation domains, which can bind to specific fragments on DNA, leading to alterations in DNA structure and transcription status [15–17]; C-end has β-catenin protein binding domain, thereby inhibiting the activity of the β-catenin/TCF transcription complex suppresses the Wnt signaling pathway [18]. SOX7 is involved in diverse developmental processes, including hematopoiesis [19], cardiovascular generation [15], endodermal differentiation [20], and myosatellite cell generation [21]. Recent studies have unveiled its tumor-suppressive role as a transcription factor in renal cell carcinoma [22], lung [23], breast [24], and prostate cancers [25]. However, the mechanism underlying the involvement of SOX7 in BCa remains unelucidated.

Globins, a protein family acknowledged for its ability to bind and transport oxygen, include prominent members like hemoglobin and myoglobin, which are pivotal for tissue oxygenation [26]. Recently, cytoglobin (CYGB), a focal point of current research within this family, has been identified as playing a unique role under certain conditions [27]. Globins are predominantly found in erythrocytes, muscle, and neural tissues; however, the expression of CYGB has been observed across diverse tissues and organs. Like other globins, CYGB possesses a compact helical structure and has the ability to reversibly bind diatomic gas molecules like NO, O_2_, and CO [28]. CYGB expression is known to increase in response to hypoxia, oxidative stress, or fibrotic stimuli. This is thought to be a mechanism that protects cells from oxidative stress damage. The abnormal expression of CYGB has been linked to various human cancers [29–31]. Investigations on Cygb^−/−^ mice have revealed a significant increase in tumors in various organs such as lungs and livers in comparison to control mice, which confirms the tumor-suppressor effect of Cygb in vivo. Furthermore, the promotor region of CYGB exhibits heightened methylation in various types of cancer cell lines and solid tumors in comparison to normal tissues and non-tumor cell lines [32]. Nonetheless, the mechanism of how the CYGB promoter is methylated and how it exerts its tumor-suppressive effects has not been fully elucidated.

DNA methylation is a crucial type of epigenetic regulation involving the transfer of reactive methyl groups to a target site without altering the composition of the DNA sequence catalyzed by methyltransferases [33]. Both the dynamics and the biological outcome of methylation are the result of the activity of a complex protein mechanism consisting of writers, erasers, and readers. DNA methylation is present in numerous tissues, and DNA that is methylated has its transcriptional processes repressed, decreasing mRNA and protein expression of genes. Many diseases are associated with DNA methylation, including the development of human cancers and their progression [34].

In this research, we reveal that SOX7 is diminished in BCa, suggesting its potential role as a tumor suppressor in this context. As an oncogene, it can lead to altered CYGB expression by downregulating the transcription of DNMT3B (DNA methyltransferase 3 beta) and the methylation level of CYGB promoter. Ultimately, the altered CYGB can affect the progression of BCa. Our findings propose SOX7 and CYGB as potential biomarkers for BCa, with implications for both its prevention and diagnosis.

## Result

### SOX7 exhibits low expression in BCa and significantly correlates with poor prognosis

**Fig. 1:**
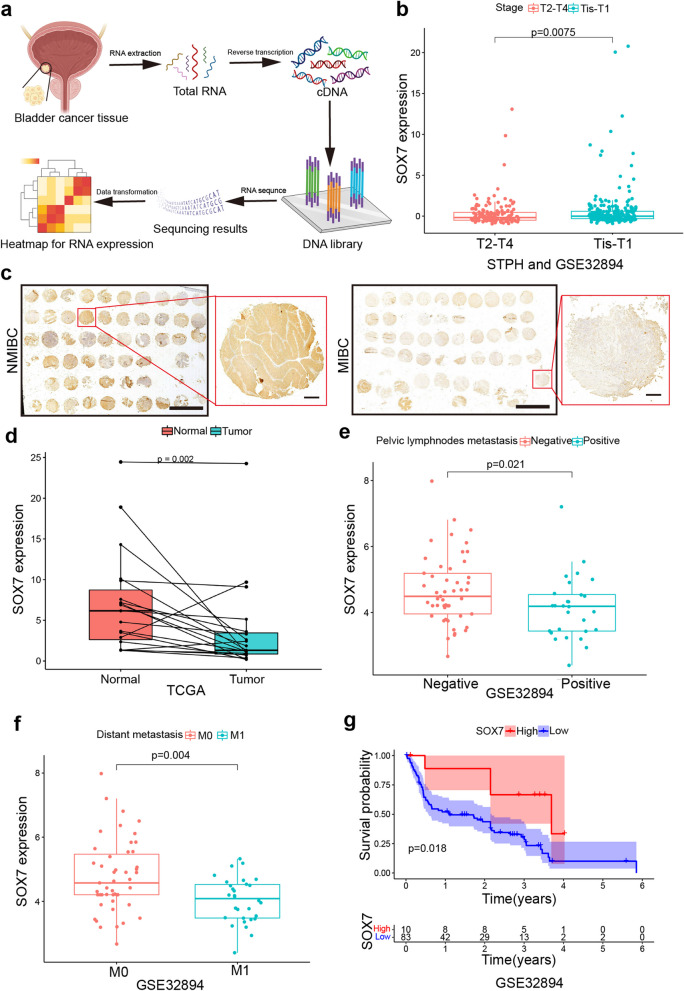
SOX7 exhibits low expression in BCa and significantly correlates with poor prognosis. **a** Flowchart of RNA-sequencing of BCa. **b** Relationship between RNA expression of SOX7 in Tis-T1 and T2-T4 at STPH and GSE32894. **c** Tissue microarray staining of BCa tissues from 61 patients about NMIBC and MIBC. Scale bars: 5 mm/400 μm. **d** RNA expression of SOX7 in normal and BCa tissues in the TCGA database. Statistical test: Paired t test. **e** and **f** RNA expression of SOX7 and its correlation with pelvic lymph nodes metastasis (**e**) and distant metastasis (**f**) in GSE32894. Statistical test: Unpaired t test. **g** The link between RNA expression of SOX7 and overall survival of individuals in the GSE32894. Statistical test: Log-rank

The RNA-sequencing of GSE32894 and 111 BCa tissues from Shanghai Tenth People's Hospital (STPH) revealed that SOX7 exhibited a significantly reduced expression in BCa tissues at T2-T4 stage than Tis-T1 (Fig. 1a and b). Tissue microarray staining of BCa tissues acquired from 61 patients was performed, and the results were scored based on histochemical staining (Fig. 1c). The acquired data revealed that individuals exhibiting low expression of SOX7 displayed heightened pathological and T-stage (T2-T4) BCa grades (Table. S1). Afterward, the acquired results underwent validation via the the Cancer Genome Atlas (TCGA) database, SOX7 exhibited significantly diminished expression in BCa tissues (Fig. 1d). Analysis of the GSE32894 dataset, often utilized to examine the link between genes and the prognosis of individuals with BCa, depicted a considerable association between the differential expression of SOX7 and the pelvic lymph nodes metastasis and distant metastasis in patients (Fig. 1e and f). Meanwhile, the acquired data implied that patients with higher SOX7 expression exhibited better prognosis (Fig. 1g).

**Fig. 2:**
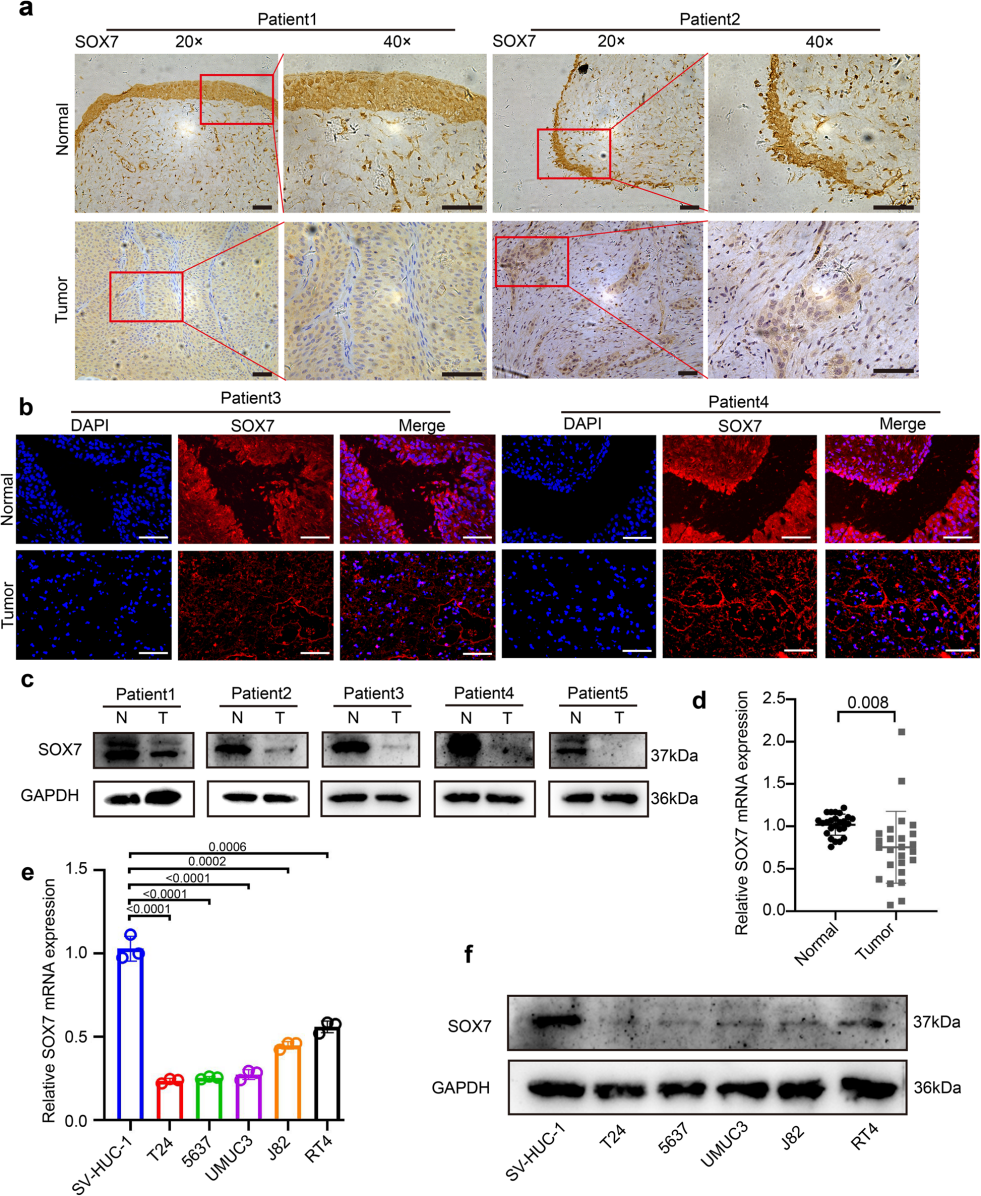
Downregulation of SOX7 in BCa tissues and cell lines. **a** IHC images illustrating the SOX7 expression in the tumor and normal tissues (*n* = 2). Scale bars: 50 μm. **b** IF images detailing the SOX7 expression in the tumor and normal tissues (*n* = 2). Scale bars: 50 μm. **c** WB describing the SOX7 expression in tumor and normal tissues (*n* = 5). **d** qPCR detection showing relative SOX7 mRNA expression in tumor and normal tissues (*n* = 25). Statistical test: Paired t test. **e** qPCR analysis displaying relative SOX7 mRNA expression in BCa cell lines. Statistical test: Unpaired t test. **f** WB illustrating SOX7 expression in BCa cell lines

The differential expression of SOX7 in BCa and normal bladder tissues was further verified through Immunohistochemistry (IHC), immunofluorescence (IF), and western blot (WB). The resulting data revealed that the expression of SOX7 in BCa tissues was markedly lower in comparison to the normal bladder tissues (Fig. 2a-c). Afterward, the variance in SOX7 transcription level in 25 pairs of cancer and normal bladder tissues was verified by qPCR, revealing a statistically significant reduction in the expression of SOX7 mRNA in BCa tissues (Fig. 2d). Additionally, this result remained consistent across the BCa cell lines. SV-HUC-1, a normal urothelial cell, served as the control. Compared with SV-HUC-1, RNA and protein levels of SOX7 in BCa cell lines T24, 5637, UMUC3, J82, and RT4 were significantly downregulated (Fig. 2e and f). The above findings are indicative of the consistent downregulation of SOX7 in BCa tissues and cells.

### SOX7 suppresses the proliferation of BCa cells in vivo

**Fig. 3:**
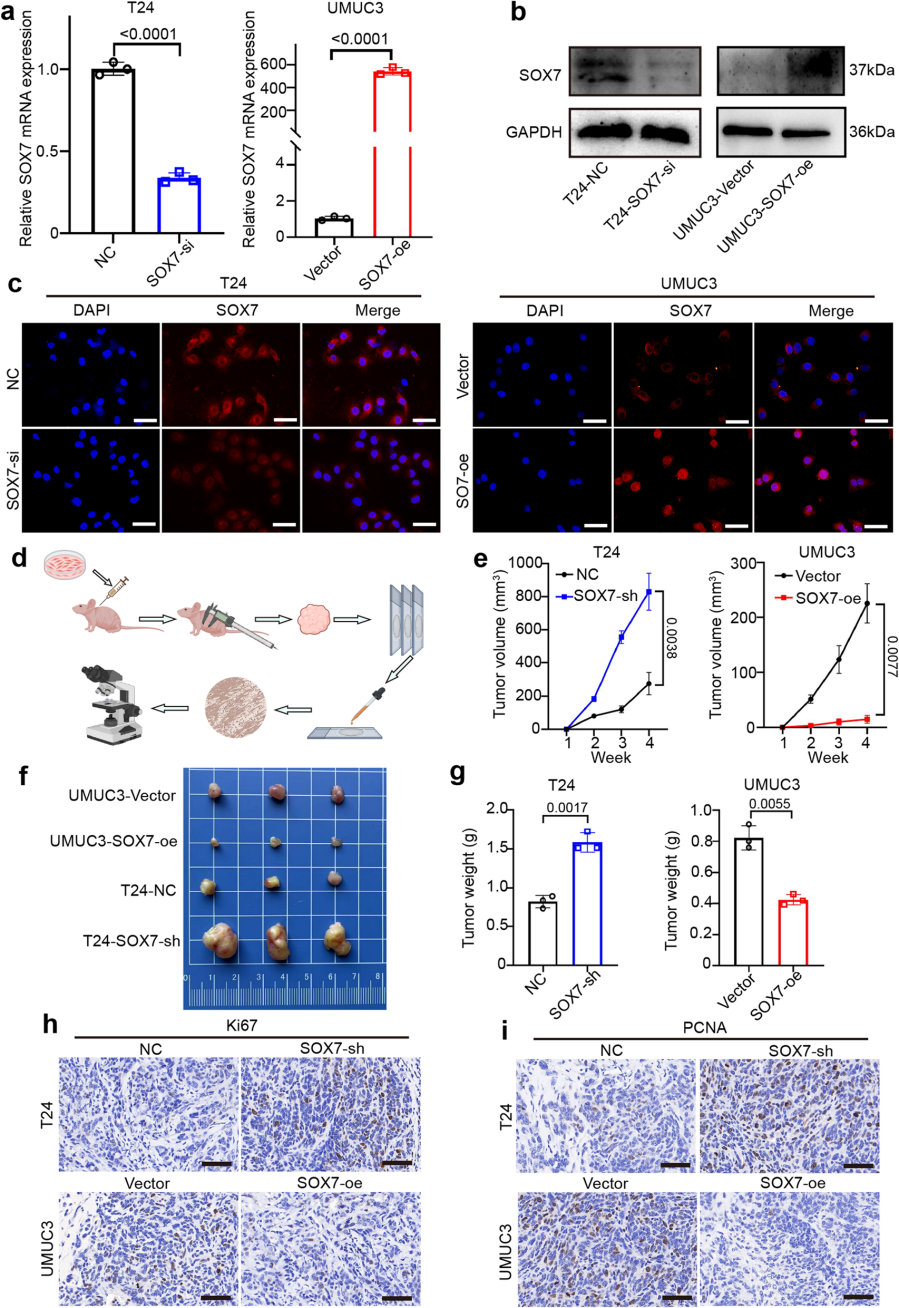
SOX7 suppresses the proliferation of BCa cells in vivo. **a**-**b** qPCR and WB detection of knockdown and SOX7 overexpression in T24 and UMUC3 cell lines. Statistical test: Unpaired t test. **c** IF images depicting knockdown and SOX7 overexpression in T23 and UMUC3 cell lines. Scale bars: 50 μm. **d** Schematic diagram of UMUC3 overexpressed with SOX7 injected into nude mice, with tumor size measured weekly. After four weeks, nude mice were euthanized, and tumors were removed, weighed, and recorded. Tissues were subsequently sectioned and stained. **e** Tumor volume growth curve. Statistical test: Unpaired t test. **f** Image of tumor size in nude mice after subcutaneous tumor loading for four weeks. **g** Histogram depicting the difference in tumor weight between the NC group and SOX7-sh group, Vector group and SOX7-oe group. Statistical test: Unpaired t test. **h**-**i** Representative IHC images of Ki67 and PCNA in mouse subcutaneous tumor tissue. Scale bars: 100 μm

To verify whether SOX7 inhibits tumor progression, SOX7 was knocked down in the T24 and UMUC3 cell lines, while SOX7 overexpression was induced in UMUC3 and T24 cell lines. The efficiency of knockdown and overexpression of SOX7 was verified at the RNA and protein levels, respectively (Fig. 3a-c, Fig. S1a-b and S2a-b). After verification, the UMUC3 cell line with overexpression and T24 cell line with stable knockdown of SOX7 (SOX7-sh) were selected for subcutaneous tumor-bearing in BALB/c-nude mice (Fig. 3d). The volume of the tumor was assessed once a week and recorded (Fig. 3e). The mice were euthanized at the fourth week, and the tumor was dissected and weighed (Fig. 3f and g). The resulting data implied that heightened expression of SOX7 was capable of considerably suppressing the proliferation of BCa in vivo. Ki67 and PCNA are markers for cell proliferation. IHC performed on the extracted tumor body highlighted that the expression of Ki67 and PCNA was negatively linked to SOX7 expression (Fig. 3h and i). The above findings underscore the inhibitory role of SOX7 in the proliferation of BCa cells in vivo.

### Overexpression and knockdown of SOX7 affect the malignant progression of BCa in vitro

**Fig. 4:**
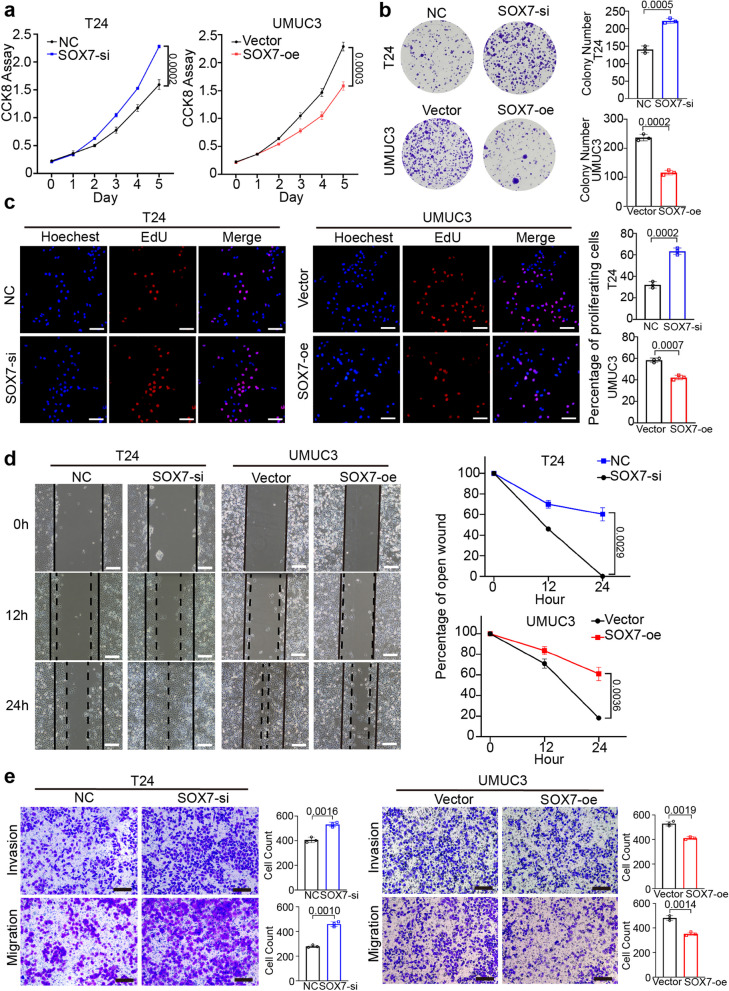
Overexpression and knockdown of SOX7 affect the malignant progression of BCa in vitro. **a**-**b** CCK-8 and colony formation assays were utilized to assess the viability of the T24 and UMUC3 cell lines following SOX7 knockdown and overexpression, respectively. Statistical test: Unpaired t test. **c** EdU assay was employed to estimate the viability of the T24 and UMUC3 cell lines following SOX7 knockdown and overexpression, respectively. Scale bars: 100 μm. Statistical test: Unpaired t test. **d** Wound-healing assay was conducted to analyze the migration abilities of T24 and UMUC3 cell lines following SOX7 knockdown and overexpression, respectively. Scale bars: 400 μm. Statistical test: Unpaired t test. **e** Transwell assay was executed to analyze the migratory and invasive capabilities of T24 and UMUC3 cell lines following SOX7 knockdown and overexpression, respectively. Scale bars: 100 μm. Statistical test: Unpaired t test

Subsequently, a series of experiments were executed to verify the impact of SOX7 on the progression of BCa. The cell counting kit-8** (**CCK-8) experiment affirmed that in comparison to the vector group, cell proliferation was markedly enhanced in the SOX7 knockdown group and diminished in the SOX7 overexpression group (Fig. 4a, Fig S1c and S2c). Similarly, EdU and colony formation experiments also showed the same outcomes (Fig. 4b-c and Fig S1d-e and S2d-e). In addition, wound healing and transwell experiments revealed that the capacity of cells to migrate and invade was enhanced after knocking down SOX7 but weakened after overexpressing SOX7 (Fig. 4d-e, Fig S1f-g and S2f-g). The acquired data are indicative of the inhibitory potential of SOX7 in the malignant progression of BCa in vitro.

### SOX7 upregulates CYGB expression in BCa

**Fig. 5:**
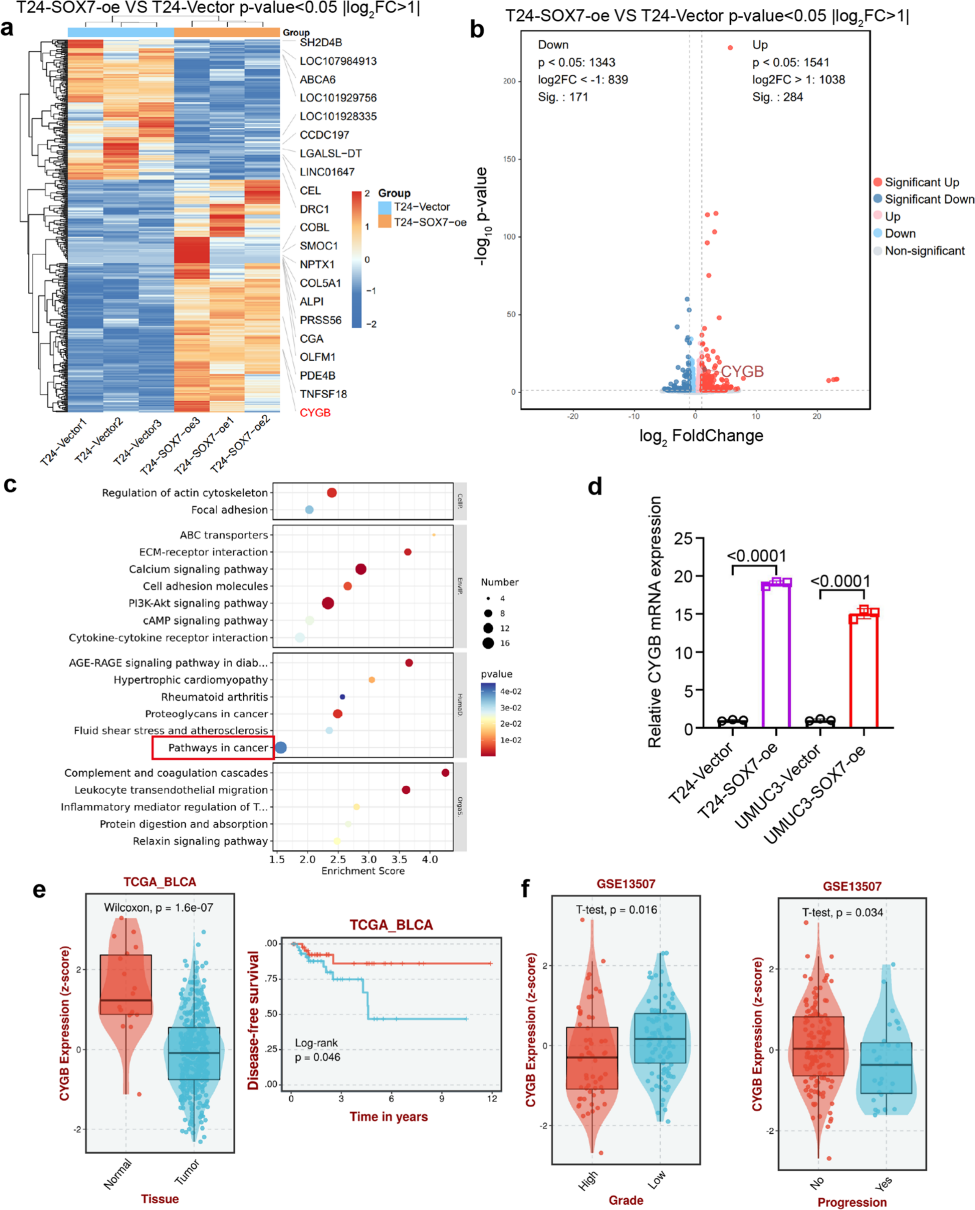
SOX7 upregulates CYGB expression in BCa. **a** Heatmaps depicting RNA-sequencing results. **b** Volcano plots illustrating RNA-sequencing data. **c** KEGG enrichment analysis of RNA-sequencing. **d** mRNA expression of CYGB in T24 and UMUC3 cell lines overexpressing SOX7. Statistical test: Unpaired t test. **e** RNA expression of CYGB in normal and BCa tissues and disease-free survival in the TCGA database. Statistical test: Wilcoxon and Log-rank. **f** Link between the RNA expression level of SOX7 and tumor grade and progression in GSE13507. Statistical test: Unpaired t test

To investigate the impact of SOX7 on the malignant progression of BCa, RNA-sequencing was conducted on the T24 cell line overexpressing SOX7. Heatmap analysis (Fig. 5a) and Volcano plots (Fig. 5b) demonstrated that the mRNA levels of CYGB were elevated alongside the increased expression of SOX7. Consistent with the findings, KEGG pathway enrichment analysis of the RNA-sequencing results revealed enrichment in pathways in cancer (Fig. 5c). Subsequently, qPCR experiments were conducted to validate these results, confirming a significant increase in CYGB mRNA levels in cells overexpressing SOX7 (Fig. 5d).

Next, exploration of CYGB expression and disease-free survival in BCa was performed using the TCGA database, revealing significantly lower expression of CYGB in BCa tissues and poorer disease-free survival (Fig. 5e). Additionally, analysis of the GSE13507 dataset indicated a correlation between CYGB expression and tumor pathological grade as well as progression (Fig. 5f). These outcomes highlight a potential oncogenic role for CYGB in BCa.

### SOX7 promotes the methylation level of the CYGB promoter by inhibiting the transcription of DNMT3B

**Fig. 6:**
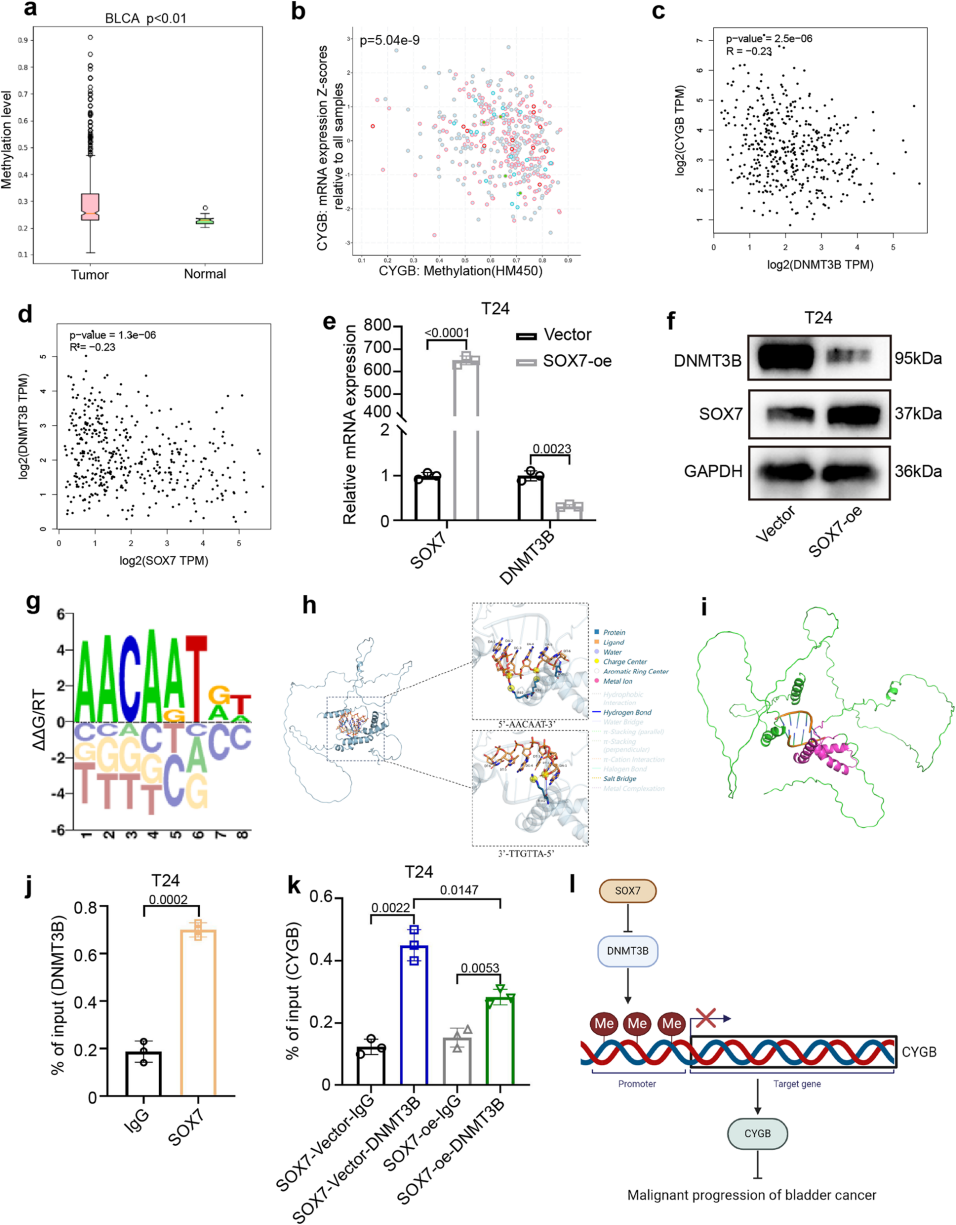
SOX7 promotes the methylation level of the CYGB promoter by inhibiting the transcription of DNMT3B. **a** Analysis results of MethHC on methylation of CYGB. Statistical test: Unpaired t test. **b** Analysis results of cBioPortal on methylation of CYGB. Statistical test: Spearman. **c** Analysis results of GEPIA on the correlation between SOX7 and DNMT3B. Statistical test: Spearman. **d** Analysis results of GEPIA on the correlation between DNMT3B and CYGB. Statistical test: Spearman. **e** Reduced expression of DNMT3B mRNA after overexpression of SOX7 in T24 cell line. Statistical test: Unpaired t test. **f** Reduced expression of DNMT3B protein after overexpression of SOX7 in T24 cell line. **g** Motif schematic of SOX7. **h** Schematic diagram of the combination of SOX7 and 5’-AACAAT-3’ in the promoter region of DNMT3B. **i** Schematic diagram of SOX7 binding to DNA: In red is the amino acid sequence 45–113 of SOX7 and the region where it binds DNA. **j** ChIP-qPCR result of DNA fragments pulled-down by SOX7 antibody in T24 cell line. Non-specific IgG was used as control. Primer was specific to region of DNMT3B promoter. Statistical test: Unpaired t test. **k** ChIP-qPCR result of DNA fragments pulled-down by DNMT3B antibody in SOX7 overexpression T24 cell line. Non-specific IgG was used as control. Primer was specific to region of CYGB promoter. Statistical test: Unpaired t test. **l** Mechanism of SOX7 affecting the malignant progression of BCa

In breast cancer, it has been noted that CYGB exhibits low expression attributed to promoter methylation [27], thereby exerting a tumor-suppressive function. Similarly, a parallel phenomenon was observed in BCa. Methylation analysis of CYGB in BCa using MethHC [35] and cBioPortal [36] revealed elevated methylation levels in BCa tissues(Fig. 6a and b). Correlation analysis from the GEPIA database unveiled a negative correlation between DNMT3B and CYGB (Fig. 6c), while SOX7 and the methyltransferase DNMT3B also displayed a significant negative correlation (Fig. 6d). Given that SOX7, as a transcription factor, can bind to the DNA promoter and may inhibit the transcription of DNMT3B. Subsequent qPCR and WB analysis confirmed reduced DNMT3B expression upon overexpression of SOX7 in the T24 cell line (Fig. 6e and f).

Motif of SOX7 was examined (Fig. 6g) [37] and protein-DNA binding prediction for the promoter region of SOX7 and DNMT3B was conducted. Remarkably, a binding site for SOX7 with the sequence 5’-AACAAT-3’ was identified in the DNMT3B promoter (Fig. 6h and i), consistent with the binding site for SOX7 reported in the UniPort database. Based on this, we designed primers targeting the DNMT3B promoter region, and the primers covered the binding site of SOX7. The results of chromatin immunoprecipitation-qPCR (ChIP-qPCR) showed that the primers were significantly enriched in SOX7 (Fig. 6j). To verify that DNMT3B regulates the methylation level of CYGB under the regulation of SOX7, ChIP- qPCR experiment on the CYGB promoter using DNMT3B antibody was conducted. The results showed that DNMT3B can bind to the CYGB promoter and its expression is regulated by SOX7 (Fig. 6k). Therefore, SOX7 reduces the methylation level of CYGB promoter by downregulating the expression of DNMT3B, thereby playing a role in inhibiting the progression of BCa following the increase in expression of CYGB (Fig. 6l).

### Construction a nomogram to prediction of prognosis of BCa patients

**Fig. 7:**
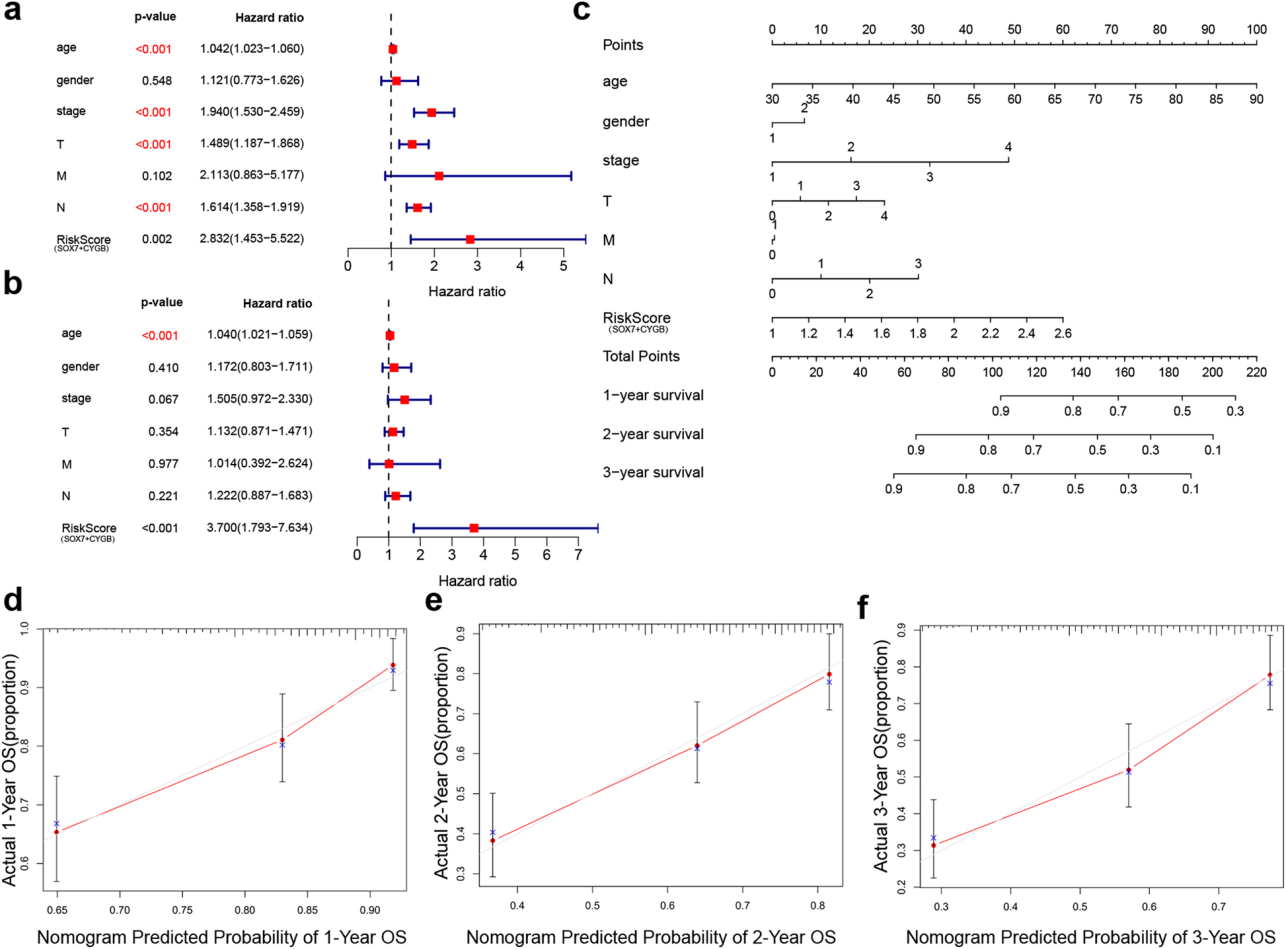
Combined prediction of prognosis of BCa patients by SOX7 and CYGB. **a** Univariate Cox regression analyses were carried out to examine variables significantly linked to OS. **b** Multivariate Cox regression analyses were carried out to examine variables significantly linked to OS. **c** Nomogram to predict the probability of 1-, 2-, and 3-year OS. **d**-**f** Calibration plots of the nomogram were utilized to predict the probability of 1-, 2-, and 3-year OS. Statistical test: Cox regression analysis (univariate and multivariate) is used to predict the predictive impact of genes and clinical pathological features

To further evaluate the clinical value of SOX7 and CYGB using the TCGA database, a combined SOX7 + CYGB scoring system was constructed through bioinformatics analysis. This combined score was identified as an independent risk factor for assessing overall survival (OS) in individuals with BCa (Fig. 7a and b). Subsequently, a nomogram was developed based on this scoring system to predict patient prognosis (Fig. 7c), and the final calibration curve demonstrated that the nomogram exhibited good predictive performance for patient OS at 1, 2, and 3 years (Fig. 7d-f).

## Discussion

In this investigation, a considerable downregulation of SOX7 expression in BCa was observed. Identified as a transcription factor, SOX7 demonstrates inhibitory effects on DNMT3B expression, thereby facilitating the upregulation of the downstream gene CYGB. Through this pathway, SOX7 plays a role in suppressing the migratory, proliferative, and invasive capacities of BCa. The findings of this research clarify the mechanism of BCa progression and can predict the prognosis of individuals with BCa through the combined score of SOX7 and CYGB.

More and more evidences suggest that SOX7 has a tumor-suppressive effect [38–40]. In many human cancers, the expression of SOX7 is downregulated [41], and overexpression of SOX7 protein can inhibit the proliferation of colon and lung cancer cells [23, 42]. When delving into the mechanism underlying SOX7 and cancer development, ample evidence supports the direct interaction of SOX7 with β-catenin binding, thereby facilitating the negative regulation of Wnt/β-catenin signaling to modulate cell growth [12, 43–45]. Nevertheless, subsequent studies have demonstrated that the anti-tumor impact of SOX7 is not solely reliant on the regulation of Wnt signaling. It has been observed that overexpression of mutant SOX7, lacking the β-catenin binding domain, still maintains the inhibitory activity of SOX7, as confirmed in colony formation experiments [28]. This indicates that in addition to Wnt signaling transduction, SOX7 may also participate in the regulation of other pathways related to cancer. Through experiments, it was found that SOX7 exhibits reduced expression in BCa. Following the overexpression of SOX7, a considerable decrease was observed in the proliferative, invasive, and migratory abilities of BCa. This underscores the tumor-suppressive role of SOX7 in BCa, akin to its effects observed in breast and lung cancers. Additionally, RNA-sequencing revealed a concurrent upregulation of CYGB upon SOX7 overexpression.

Recent evidence indicates that CYGB exerts anti-tumor effects across various human malignancies. In this research, a reduction in CYGB expression was observed in tissues linked to ovarian, head and neck, liver, lung, esophageal, pancreatic, and breast cancers [29, 46–53]. Moreover, diminished CYGB expression has been reported in individuals with glioma, correlating with elevated histological grade and increased risk of tumor recurrence [54]. Moreover, investigations into liver cancer development revealed that CYGB-deficient mice exhibited heightened susceptibility to liver cancer induction in both chemically induced and high-fat diet models [31]. In breast cancer, researchers have identified that CYGB inhibits breast cancer by suppressing glucose metabolism [27]. Collectively, these discoveries imply an anti-tumor role for CYGB. Similarly, this investigation highlights that CYGB, as a downstream target of SOX7, plays a tumor inhibitory function with SOX7 in BCa. Analyzing from a transcriptomic perspective, CYGB is highly expressed in normal BCa tissues, and patients with BCa with lower CYGB expression have poorer DFS, higher tumor grade, and poorer progression. Furthermore, our results indicate hypermethylation of the CYGB promoter in BCa.

Many genes are hypermethylated at their promoters and function as oncogenes in tumor development. However, upon restoration of their expression, these genes have the potential to impede tumor initiation and/or progression [55, 56]. Consistent with evidence demonstrating the epigenetic downregulation of CYGB in several types of malignancies [57, 58], demethylation treatment effectively restored CYGB expression. This confirms that promoter methylation participates in the suppression of CYGB expression across a wide range of cancers. As a member of the DNA methyltransferase family, DNMT3B has the capacity to enhance methylation on promoters of downstream genes, consequently suppressing the transcription process of these genes. Our analysis of the BCa database suggests a potential association between hypermethylation of CYGB and the malignant progression of BCa, which could be attributed to DNMT3B-mediated hypermethylation of the CYGB promoter.

In summary, this study reveals that SOX7 expression is downregulated in BCa, which has implications for regulating CYGB expression through the suppression of DNMT3B transcription and subsequent reduction methylation levels of CYGB promoter. Consequently, these changes in CYGB expression may impact the progression of BCa. Additionally, the combined SOX7 + CYGB score holds promise for predicting the prognosis of BCa patients. Nonetheless, our study has certain limitations. Such as a larger sample size is required to supplement and validate the developed scoring system. Although we analyzed the role of CYGB in BCa from the perspective of transcriptomics, the functional correlation of CYGB in BCa cells still needs further experimental verification.

## Materials and methods

### Data retrieval

The BCa dataset accessed at STPH, as well as The Cancer Genome Atlas (cancergenome.nih.gov/TCGA) and Gene Expression General Database (https://www.ncbi.nlm.nih.gov/GEO/), were used in this study. The STPH dataset comprised 111 patients who fulfilled the following three inclusion criteria: (1) The histological diagnosis confirmed by pathologists was BCa; (2) The prognostic information was complete and could be used for analytical studies; (3) RNA expression data met the available criteria. From November 2019 to April 2023, 111 patients with BCa were recruited from STPH data. All studies, including human participants, underwent review and approval by the STPH Ethics Committee (approval number 2021KN108). Since this investigation is retrospective in nature, informed consent of patients was not essential. Total RNA extraction, bilateral library generation, and RNA sequencing protocols for STPH were the same as established within our previously published studies [59].

### Cell lines and cell culture

The human BCa cell lines utilized in this study included T24, 5637, UMUC3, J82, and RT4. Immortalized human normal bladder epithelial cell line SV-HUC-1 from the Chinese Academy of Sciences (Shanghai, China) was also used. SV-HUC-1 was cultured in F12k medium (Sigma-Aldrich, St. Louis, MO, USA), whereas RPMI-1640 medium (Thermo Fisher Science, Inc. USA) was utilized to culture T24, 5637, and UMUC3 cell lines. RT4 cell lines were grown in McCoy's 5A medium (Thermo Fisher Science, Inc. USA). The culture media utilized in these processes was enriched with 1% penicillin/streptomycin (Hyclone, Logan, UT, USA) and 10% fetal calf serum (FCS; Thermo Fisher Scientific, Inc. USA). The culturing of these cell lines was implemented in an incubator under 5% CO_2_ at a constant temperature of 37° C.

### Western blot

Cells or tissues underwent lysis with RIPA (Beyotime, China) for 30 min, with subsequent centrifugation at 12,000 rpm for 10 min. The supernatant was taken, and total protein concentration was analyzed using Bicinchoninic Acid protein assay (BCA; Beyotime, China). Prior to Western blot analysis, protein samples (40 μg each) were subjected to electrophoresis utilizing 10% sodium dodecyl Sulfate–polyacrylamide gel (SDS-PAGE) with subsequent transfer to nitrocellulose membranes (Sigma-Aldrich; Merck KGaA). For blocking these membranes, a solution comprising PBS + 5% skim milk was utilized for 1 h, with subsequent overnight exposure to primary antibodies at 4° C. The membranes then underwent washing with PBST (thrice) and were exposed to horseradish peroxidase-coupled secondary antibodies at room temperature for 1 h. Detection of labeled proteins and determination of their concentration was carried out via a chemiluminescence imaging system (Tanon 5200 system, Tanon, Shanghai, China). Antibodies were shown at Supplementary information.

### RNA isolation and quantitative real-time PCR (qPCR)

The extraction of total RNA from human tissues or cells was carried out utilizing the TRIzol reagent. The first cDNA strand was then generated utilizing a reverse transcription system kit (Vazyme Biotech Co., Ltd., China). Afterward, qPCR was performed utilizing the ABI Prism 7500 sequence detection system (Applied Biosystems, CA) and ChamQ Universal SYBR qPCR Master Mix (Vazyme, China).

The following parameters were established for qPCR: 95° C 5 min, 40 cycles (95° C 10 s, 60° C 30 s). The endogenous control was established as GAPDH. The relative folding changes were then determined in triplicate using the 2-^ΔΔCT^ technique. Primers were shown at Supplementary information.

### Lentivirus infection and cell transfection

Stable overexpression and knockdown of SOX7 in BCa cells was achieved through lentivirus infection. Initially, the plasmid was cloned into a lentiviral vector with subsequent transfection into HEK-293 T cells. The BCa cells underwent infection with the virus, and stable cell lines were established through selection with puromycin.

The cell transfection process was carried out utilizing 1 × 10^5^ BCa cells/well inoculated into a 6-well plate. Then, siRNA or plasmid was transfected into cells utilizing lipofectamine 3000 (Invitrogen, USA) following the provided instructions. The efficiency of the transfection process was assessed via qPCR and Western blot techniques.

### Nude mice xenograft assay

This assay involved subcutaneous transplantation of the tumor. Specifically, subcutaneous administration of 5 × 10^7^ BCa cells was carried out into the right axilla of 4-week-old BALB/c-nude mice. After a 4-week period, the animals were euthanized, and the excised tumor was utilized for further follow-up experiments. Throughout the experimental period, the volume and diameter of the tumor, as well as the body weight of the mice, were monitored after a 7-day interval. Tumor volume was determined via the given formula: Tumor volume = π/6 × length × width^2^ [59].

### Immunohistochemistry and immunofluorescence analysis

Fresh tissue was removed and underwent fixation in cold 4% paraformaldehyde (PFA), with subsequent embedding in paraffin blocks 48 h later. Following this process, the tissues were then sectioned. The sections were then de-paraffinized, graded alcohol dehydrated, antigen repaired, and blocked with 3% BSA in xylene for 1 h. After the completion of this process, incubation of these tissue sections was carried out with antibodies at 4° C for 18 h. The sections then underwent exposure to biotinylated rabbit anti-goat IgG for 20 min at 25° C, with subsequent exposure to streptavidin–horseradish peroxidase or fluorescent peroxidase for 30 min. Diaminobenzidine-H_2_O_2_ and hematoxylin were then used for tissue staining (this step is not required for IF).

The tissues were selected and grouped into MIBC and NMIBC for microarray. We regularly conduct clinical follow-up on patients and results have been published [60]. Followed by statistical analysis for group comparison using Pearson's chi-squared test.

### Cell counting kit-8 assay

The degree of cell proliferation was detected utilizing the CCK-8 assay (Yeasen, Shanghai, China). For this purpose, 96-well plates were utilized for seeding 500 cells/wells. The next step involved introducing 10 μl of CCK-8 reagent into each well on days 0, 1, 2, 3, 4, and 5 of inoculation, respectively, with subsequent incubation of all samples at 37° C for 1.5 h. A microplate spectrophotometer (BioTek instrument, Winooski, VT) was utilized to examine the absorbance of the samples at 450 nm in order to determine cell proliferation.

### Colony formation assay

For colony formation assays, 1 × 10^3^ cells/wells were seeded into a 6-well plate and left for nine days. The 6-well plate was then rinsed with PBS (thrice), followed by fixation via 4% PFA. The cell community was then stained with 0.1% crystal violet (Yeasen, Shanghai, China) for 20 min, rinsed with PBS, and dried for 30 min. Finally, the cell colony was photographed with a digital camera.

### EdU incorporation assay

The cells were seeded into 96-well plates at a density of 1 × 10^3^ cells/well and evaluated for cell proliferation after 24 h using the EdU assay kit (K1077, APExBIO Corporation, US). The stained cells were then imaged using fluorescence microscopy (Nikon, Tokyo, Japan).

### Wound healing assay

For the wound-healing assay, cells were cultured in 6-well plates, and cell layers were scratched by a sterile plastic suction pipette. Subsequent imaging was conducted using electron microscopy (Nikon, Tokyo, Japan) at 0, 12, and 24 h, respectively. The migratory capacity of cells was evaluated by assessing changes in the size of the injured area.

### Migration and invasion assays

Transwell film with or without Matrix (Yeasen, Shanghai, China) was utilized for evaluating the invasive or migratory ability of BCa cells. Specifically, the chamber was placed into a 24-well plate, and 2.5 × 10^4^ cells were inoculated into the upper chamber, containing 200 μl FCS-deficient medium. At the same time, 600 μl of culture medium with 10% FCS was introduced to the lower chamber. Following incubating at 37 ℃ for 24 h, the culture chamber was rinsed with PBS and fixed in 4% paraformaldehyde for about 15 min. The next step involved scraping off the cells on the upper side of the membrane with a cotton swab and staining via crystal violet at room temperature for about 15 min. Subsequently, the film was rinsed with PBS and photographed after drying out.

### RNA-sequencing analysis

In this experiment, cells overexpressing SOX7 and negative control cells were extracted with subsequent isolation of the total RNA. An Illumina HiSeq 2000 (Shanghai OE Biotechnology Co., Ltd.) was utilized to conduct the RNA-seq procedure. The STAR aligner v2.5 was then utilized to comparatively assess the retrieved data with the UCSC human genome (hg19), and the relevant hits underwent quantification using feature counting software. The RNA-seq data were then subjected to analysis via "DESeq2" and "ClusterProfiler" software. The cut-off values were established as a multiplicity of change of 2 and an adjusted *p*-value of 0.01.

### Protein-DNA molecular docking

The AlphaFold-predicted protein 3D structure file SOX7_HUMA (UniProt ID: Q9BT81) was downloaded from UniProt, and the DNA (5'-AACAAT-3') 3D structure was constructed using Discovery Studio 2019. HDOCK is designed to predict binding complexes between two molecules, including proteins and nucleic acids, by employing a hybrid docking strategy. Protein-DNA molecular docking was applied by setting appropriate docking parameters using protein as receptor and DNA as ligand.

### Nomogram establishment and validation

Cox regression analyses (univariate and multivariate) were performed to predict the predictive impact of SOX7 + CYGB and other clinicopathological attributes. Factors that exhibited significance in multivariate Cox regression analyses were subsequently used to create nomograms, which underwent validation via the "Rms" R package v5.1 (https://cran.r-project.org/web/packages/rms/index.html). Validation was conducted by means of the consistency index (C-index) and the calibration graphs.

### Chromatin immunoprecipitation assay

ChIP assays were conducted using the SimpleChIP Enzymatic Chromatin IP Kit (Cell Signaling Technology, MA, USA). The cells were crosslinked with 4% polyformaldehyde (Biosharp, Anhui, China) for 10 min, followed by treatment with glycine. After washing with cold PBS, the cells were lysed and the DNA was digested with micrococcal nuclease into 150–900 bp fragments. These fragments were immunoprecipitated overnight at 4 °C using antibodies against the specific protein or IgG control. The ChIP-enriched DNAs were then analyzed by qRT-PCR, and the data were normalized to the input.

### Statistical analysis

All statistical analyses were conducted using SPSS software, and the results were plotted using GraphPad Prism 8 software. Shapiro–Wilk test was used to verify the normal distribution. Pairs of groups were compared using Student's t-test. Pearson's correlation analysis was employed to calculate the correlation between continuous variables. χ^2^ tests were used to analyze count data, and Fisher's exact test was applied when the sample size was less than 40. Cox regression analysis (univariate and multivariate) is used to predict the predictive impact of genes and clinical pathological features, in order to reduce the interference and bias of confounding variables on the analysis results. Error bar means standard deviation (SD).

## Supplementary Information

Supplementary Material 1.

## Data Availability

All data are available from the corresponding authors upon reasonable request.
