# Supplementary material for: SOX7 inhibits the malignant progression of bladder cancer via the DNMT3B/CYGB axis
Source: Mol Biomed. 2024 Sep 4;5:36. doi: 10.1186/s43556-024-00198-8 (PMC11371982; doi:10.1186/s43556-024-00198-8)
Supplement: Supplementary file 1 — Supplementary Material 1. [file 43556_2024_198_MOESM1_ESM.docx]

**Supplementary Information**

SOX7 inhibits the malignant progression of bladder cancer via the DNMT3B/CYGB axis

Jingcheng Zhang^1,2#^, Wentao Zhang^1,2#^, Ji Liu^1,2#^, Yuchao Liu^1,2^, Yufeng Jiang^2,3^, Ailiyaer Ainiwaer^1,2,4^, Hanyang Chen^1,2^, Zhuoran Gu^1,2^, Haotian Chen^1,2^, Shiyu Mao^1,2^, Yadong Guo^1,2^, Tianyuan Xu^1,2^, Yunfei Xu^1,2*^, Yuan Wu^5*^, Xudong Yao^1,2*^, Yang Yan^1,2*^

1 Department of Urology, Shanghai Tenth People’s Hospital, School of Medicine, Tongji University, Shanghai, China.

2 Urologic Cancer Institute, School of Medicine, Tongji University, Shanghai, China.

3 Department of Urology, Chongming Branch, Shanghai Tenth People’s Hospital, School of Medicine, Tongji University, Shanghai, China.

4 Department of Urology, Kashgar Prefecture Second People's Hospital, Kashgar, Xinjiang Uygur Autonomous Region, China

5 Department of Urology, Hefei Cancer Hospital, Chinese Academy of Sciences, Hefei, China.

# These authors have contributed equally to this work and share the first authorship.

* Author to whom correspondence should be addressed.

Corresponding author

Yang Yan: 1500069@tongji.edu.cn

Xudong Yao: yxd@tongji.edu.cn

Yuan Wu: [13956051264@163.com](mailto:13956051264@163.com)

Yunfei Xu: xuyunfeibb@sina.com


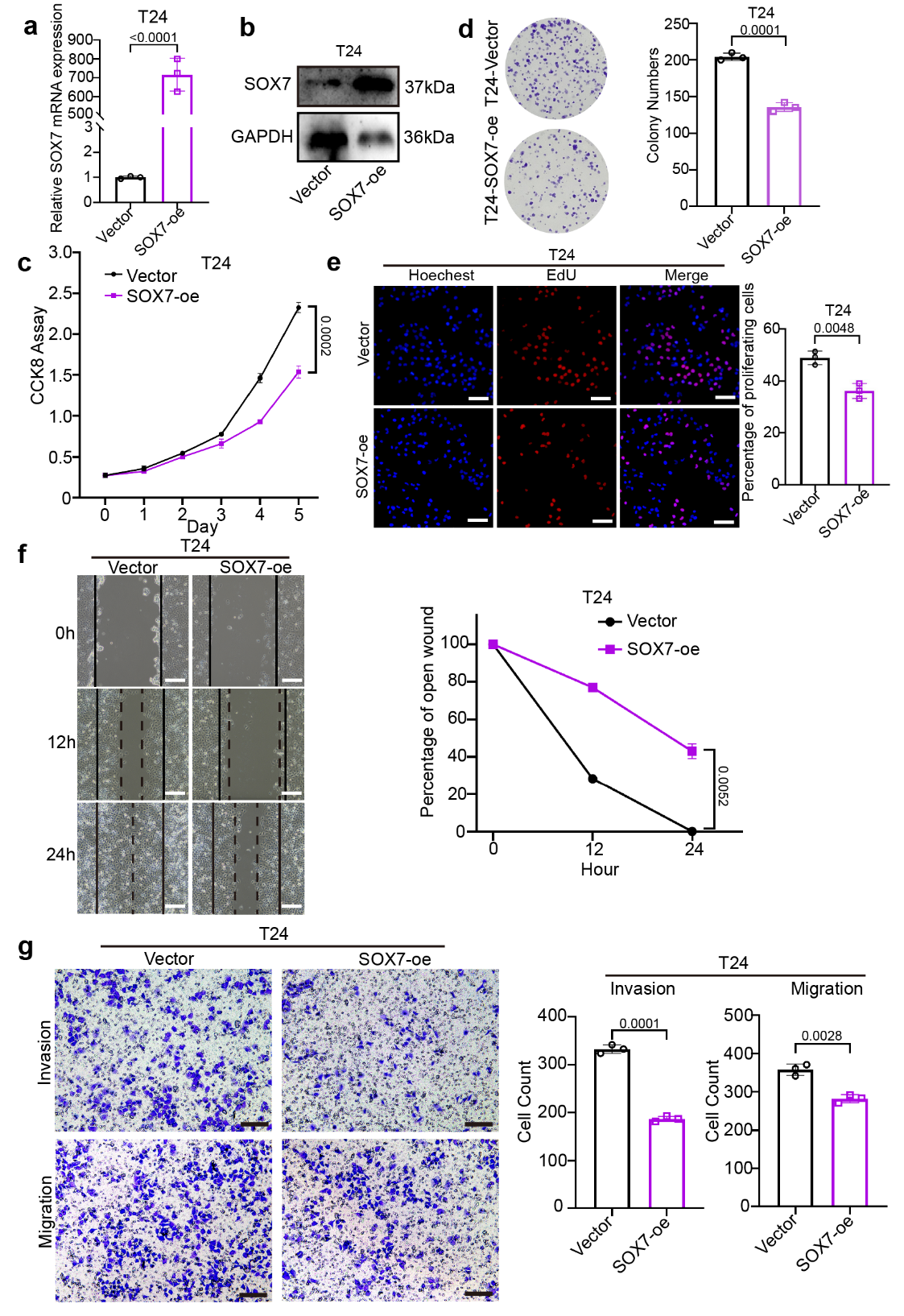


Figure S1: The impact of overexpression of SOX7 on T24 cell lines. (a-b) qPCR and western blot detection SOX7 overexpression in T24 cell line. Statistical test: Unpaired t test. (c-d) Colony formation assays and CCK-8 were utilized to assess the viability of the T24 cell line after SOX7 overexpression. Statistical test: Unpaired t test. (e) EdU assay was utilized to estimate the viability of the T24 cell line following SOX7 overexpression. Scale bars: 100μm. Statistical test: Unpaired t test. (f) Wound-healing assay was conducted to analyze the migratory capability of the T24 cell line after SOX7 overexpression. Scale bars: 400μm. Statistical test: Unpaired t test. (g) Transwell assay was executed to examine the migratory and invasive capabilities of the T24 cell line after SOX7 overexpression, respectively. Scale bars: 100μm. Statistical test: Unpaired t test.


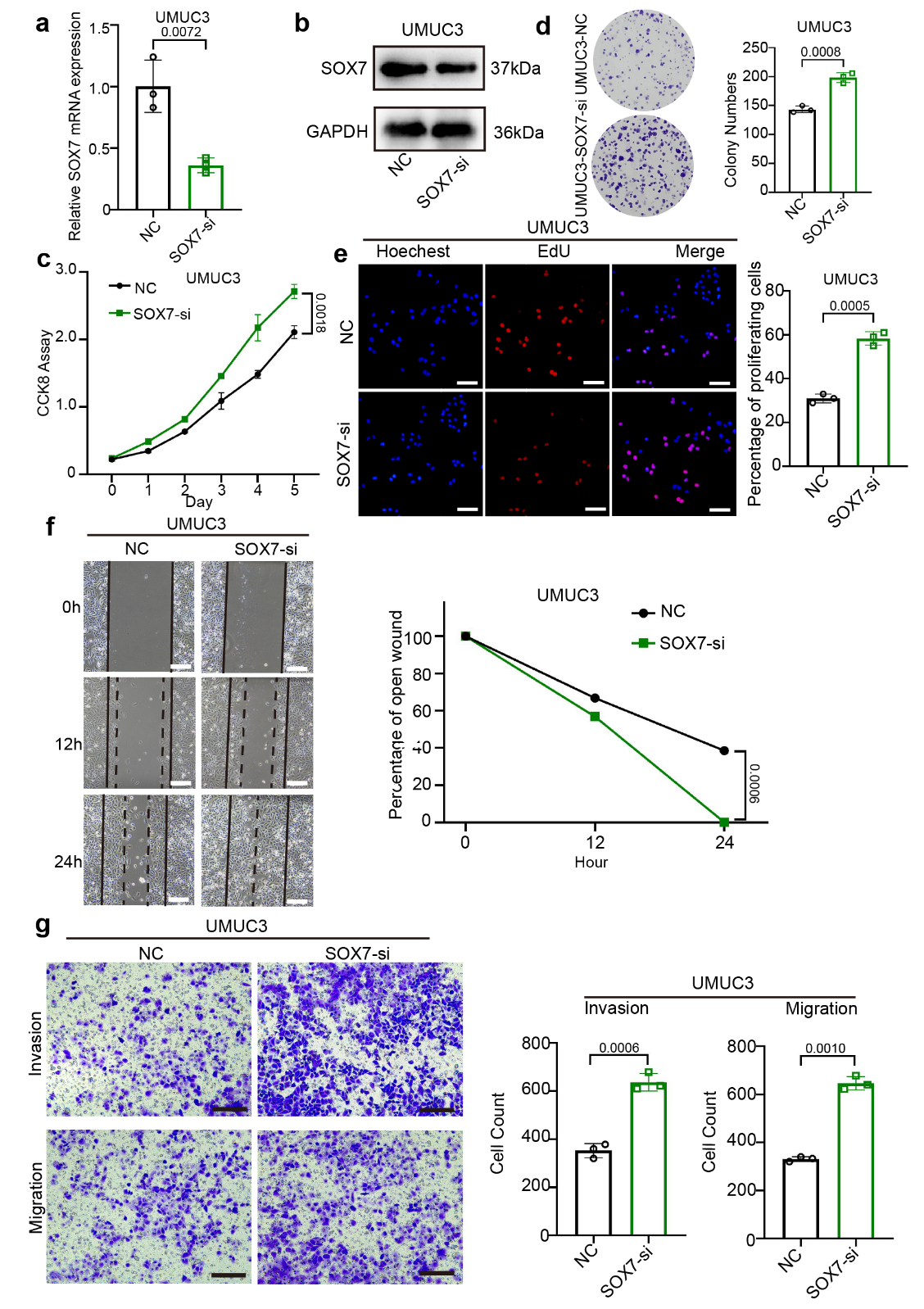


Figure S2: The impact of knockdown of SOX7 on UMUC3 cell lines. (a-b) qPCR and western blot detection SOX7 knockdown in UMUC3 cell line. Statistical test: Unpaired t test. (c-d) Colony formation assays and CCK-8 were utilized to assess the viability of the UMUC3 cell line after SOX7 knockdown. Statistical test: Unpaired t test. (e) EdU assay was utilized to estimate the viability of the UMUC3 cell line following SOX7 knockdown. Scale bars: 100μm. Statistical test: Unpaired t test. (f) Wound-healing assay was conducted to analyze the migratory capability of the UMUC3 cell line after SOX7 knockdown. Scale bars: 400μm. Statistical test: Unpaired t test. (g) Transwell assay was executed to examine the migratory and invasive capabilities of the UMUC3 cell line after SOX7 knockdown, respectively. Scale bars: 100μm. Statistical test: Unpaired t test.

Table S1: Correlation between clinicopathological features and expression of SOX7 in 61 patients with bladder cancer.

| Parameters | Low expression of SOX7 | High expression of SOX7 | p value |
| --- | --- | --- | --- |
| Gender |  |  | 0.77 |
| Male | 25 | 26 |  |
| Female | 6 | 4 |  |
| Age (years) |  |  | 0.232 |
| ≤65 | 15 | 10 |  |
| ＞65 | 16 | 20 |  |
| BMI |  |  | 0.71 |
| ≤24 | 18 | 16 |  |
| ＞24 | 13 | 14 |  |
| Histologic grade | |  | 0.028 |
| Low | 0 | 6 |  |
| High | 31 | 24 |  |
| T stage |  |  | 0.043 |
| Tis-T1 | 6 | 13 |  |
| T2-T4 | 25 | 17 |  |
| N stage |  |  | 0.212 |
| N0 | 26 | 29 |  |
| ＞N0 | 5 | 1 |  |
| M stage |  |  | 1 |
| M0 | 30 | 30 |  |
| M1 | 1 | 0 |  |
| Tumor size (cm) | |  | 0.03 |
| ＞3.5 | 21 | 12 |  |
| ≤3.5 | 10 | 18 |  |
| hypertension |  |  | 0.252 |
| Yes | 12 | 16 |  |
| No | 19 | 14 |  |
| diabetes |  |  | 0.198 |
| Yes | 5 | 9 |  |
| No | 26 | 21 |  |

Statistical tests: Pearson's correlation analysis was employed to calculate the correlation between continuous variables. χ2 tests were used to analyze count data, and Fisher's exact test was applied when the sample size was less than 40.

Table S2: Primers

| Primer | Sequence (5'-3') |
| --- | --- |
| GAPDH |  |
| Forward | GCCAAGGTCATCCATGACAACTTTGG |
| Reverse | GCCTGCTTCACCACCTTCTTGATGTC |
|  |  |
| SOX7 |  |
| Forward | TCGACGCCCTGGATCAACT |
| Reverse | CTGGGAGACCGGAACATGC |
|  |  |
| DNMT3B |  |
| Forward | AGGGAAGACTCGATCCTCGTC |
| Reverse | GTGTGTAGCTTAGCAGACTGG |
|  |  |
| CYGB |  |
| Forward | CTGTCGTGGAGAACCTGCAT |
| Reverse | GAGGGTCTTCAGAACTCGGC |

Table S3: Antibodies

| Antibodies | Product number | Company | County |
| --- | --- | --- | --- |
| GAPDH | S30201 | Yeasen | China |
| SOX7 | AF2766 | R&D | US |
| DNMT3B | 26971-1-AP | ProteinTech | China |
| Ki67 | WL01384a | Wanleibio | China |
| PCNA | A0264 | ABclonal | China |
| HRP-Goat | SA00001-4 | ProteinTech | China |
| HRP-Rabbit | ab6721 | abcam | US |
| HRP-Mouse | ab6728 | abcam | US |
